# Hematological profiles of visceral leishmaniasis patients before and after treatment of anti-leishmanial drugs at University of Gondar Hospital; Leishmania Research and Treatment Center Northwest, Ethiopia

**DOI:** 10.1186/s12879-021-06691-7

**Published:** 2021-09-26

**Authors:** Elias Shiferaw, Fadil Murad, Mitikie Tigabie, Mareye Abebaw, Tadele Alemu, Sefanit Abate, Rezika Mohammed, Arega Yeshanew, Fitsumbrhan Tajebe

**Affiliations:** 1 Department of Hematology and Immunohematology, School of Biomedical and Laboratory Sciences, College of Medicine and Health Sciences, University of Gondar, P.O. Box 196, Gondar, Ethiopia; 2 Laboratory Technologist at University of Gondar Comprehensive Specialized Hospital, Gondar, Ethiopia; 3 Department of Internal Medicine, University of Gondar Comprehensive Specialized Hospital, Gondar, Ethiopia; 4 Laboratory Technologist at University of Gondar Leishmania Research and Treatment Centre, Gondar, Ethiopia; 5 Department of Immunology and Molecular Biology, School of Biomedical and Laboratory Sciences, College of Medicine and Health Sciences, University of Gondar, Gondar, Ethiopia

**Keywords:** **-**Hematological parameter, Visceral leishmaniasis, Anti- leishmanial drugs, Gondar, Northwest Ethiopia

## Abstract

**Background:**

Visceral leshimaniasis is a parasitic disease characterized by systemic infection of phagocytic cells and an intense inflammatory response. The progression of the disease or treatment may have an effect on hematological parameters of these patients'. Thus, the current study sought to compare the hematological profiles of visceral leishmaniasis patients before and after treatment with anti-leishmaniasis drugs.

**Method:**

An institutional-based retrospective cohort study was conducted among visceral leishmaniasis patients admitted to the University of Gondar comprehensive specialized referral hospital leishmaniasis research and treatment centre between September 2013 and August 2018. Hematological profiles were extracted from the laboratory registration book before and after treatment. Data were entered to Epi-info and exported to SPSS for analysis. Descriptive statistics were summarized using frequency and percentage to present with the table. The mean, standard deviation, median, and interquartile range were used to present the data. Furthermore, using the paired t-test and the Wilcoxon Signed rank test, the mean difference for normally and non-normally distributed data was compared. Spearman and Pearson correlation analysis were used to describe the relationship between hematological parameters and various variables. A P value of 0.05 was considered statistically significant.

**Result:**

With the exception of the absolute neutrophil count, all post-treatment hematological parameters show a significant increase when compared to pre-treatment levels. Prior to treatment, the prevalence of anemia, leukopenia, and thrombocytopenia was 85.5, 83.4, and 75.8%, respectively, whereas it was 58.3, 38.2, and 19.2% following treatment. Furthermore, parasite load was found to have a statistically significant negative correlation with hematological profiles, specifically with white blood cell and red blood cell parameters.

**Conclusion:**

According to our findings, patients with visceral leishmaniasis had improved hematological profiles after treatment. The effect of treatment on parasite proliferation and concentration within visceral organs, in which the parasite load could directly affect the patient's hematological profiles, may be associated with the change in hematological profiles.

## Background

Leishmaniasis is a category of tropical parasitic diseases caused by species of the genus Leishmania and transmitted by female phlebotomine sand flies. It's a zoonotic and anthroponotic disease, with humans and dogs being the most prevalent hosts [1–3]. The disease comes in a variety of forms, with cutaneous leishmaniasis, Visceral Leishmaniasis (VL), and mucocutaneous leishmaniasis being the most common. Visceral Leishmaniasis, commonly known as Kala-Azar, is the most serious form of leishmaniasis, and it is almost invariably fatal if left untreated. It is the most common kind in Ethiopia and Eastern Africa [4]. It's caused by the leishmania donovani complex, which hangs around in the spleen and bone marrow to spread [5]. About 90% of VL patients do not acquire the disease's characteristic symptoms and are classified as subclinical or asymptomatic. When an infection advances to illness, it results in spleen and liver enlargement [6].

The parasite is found in macrophage phagolysosomes in the liver, spleen, bone marrow, and lymph nodes. The absence of delayed type hypersensitivity reactions to leishmania antigen in patients with active VL suggested a severe reduction of the cellular immune response. Macrophages are the only cells that allow leishmania parasites to develop in vivo, resulting in parasite-specific T-cells producing gamma interferon [7].

Hematological profile dysregulation has been linked to VL patients, and this could be a substantial source of mortality and morbidity. The most common hematological symptoms include anemia, leukopenia, thrombocytopenia, and pancytopenia. As a result of neutropenia, changes in hematological profiles have been linked to bleeding disorders as well as increased host susceptibility to bacterial infection [8].

Anemia has been linked to Red Blood Cell (RBC) hemolysis, dietary inadequacies, the existence of additional co-morbidities such chronic illness, and opportunistic infection [9]. Anemia and RBC morphological abnormalities can be caused by a variety of factors, including sequestration and death of RBCs in an enlarged spleen, immunological mechanisms, or changes in RBC membrane [8].

Since amastigote lives and proliferates in the mononuclear phagocytic system, namely the spleen, liver, and marrow, the severity of hematological abnormalities is determined by the duration of the disease and the size of the spleen. This causes hyperplasia of the mononuclear phagocytic system, resulting in phagocyte-bearing organs such as the spleen becoming enormously expanded and resulting in hematological symptoms [10].

Improvements in hematological profile are expected within 2 weeks of starting anti-leishmaniasis medicines, with complete recovery taking 4 to 6 weeks. Treatment methods for leishmaniasis vary, with a broad range of first-line medications including Sodium Stibogluconate (SSG) and Paromomycin in combination, Sodium Stibogluconate or Meglumin Antimoniate as Monotherapy, and Liposomal Amphotericin B. Second-line medications, on the other hand, include Liposomal Amphotericin B (AmBisome), Miltefosine, and Paromomycin. In the treatment of leishmaniasis patients, a total dose of 20 mg/kg was found to be beneficial [11].

Despite the fact that only a few studies have been published, a comprehensive understanding of changes in hematological profiles in VL patients is still lacking, which is crucial for the early detection and prevention of problems. As a result, the current study was created with the goal of evaluating VL patients' hematological profiles before and after treatment.

## Methods

### Study design, and setting

From February to March 2019, an institutional based retrospective cohort study was conducted at the University of Gondar comprehensive specialized referral hospital, leishmaniasis research and treatment center. The hospital is located in Gondar town, Amhara regional state, 738 km from Addis Ababa, Ethiopia's capital. The population of Gondar was reported to be 207,044 people in the 2007 Ethiopian census [12]. In the field of leishmaniasis, the center serves as a diagnostic, therapeutic, and research facility.

### Populations

The medical records of 463 microscopically confirmed VL patients were examined. In the current study, all patients who were admitted to the hospital between 2013 and 2018 were included.

### Data collection

A data extraction sheet was used to collect data on socio-demographic and clinical parameters such as age, sex, aspiration location, and treatment completion date. In addition, laboratory investigations, primarily parasite load and hematological parameters (RBCs count, Hb, Hct, RBCs indices, WBCs count, WBCs differential count, and platelet count) were retrieved from the laboratory registration book prior to treatment initiation (Day 0) and after treatment completion (Day 17 or 28). The completeness and consistency of the data were checked to determine the data's quality. Patients with missing clinical as well as laboratory parameters, mainly parasite load and hematological parameters were excluded from the study.

### Assessment of hematological parameters

Hematological profile had been operationally defined on the bases of reference range from the study area; Gondar, Northwest Ethiopia [13] which states Anemia: a decrease in Hb value (Hb value < 11.5 g/dl for males and < 11 g/dl for females), Leukopenia: is characterized by a reduction in the number of white blood cells (WBC count of less than 3.2*103/l), Neutropenia: decreased number of neutrophil count (Neutrophil count < 1600/µl), Neutrophilia: higher neutrophil count (> 5100/l), Lympopenia: is characterized by a reduction in the number of lymphocytes in the body (Lymphocyte count 1000/l); Lympocytosis: is characterized by an increase in lymphocyte count (> 3500/l), Parasite grade: the number of parasite per microscopic fields in bone marrow or spleen aspirate was graded as follows: (6 + : > 100 parasites/ field, 5 + : 10–100 parasites/ field, 4 + : 1–10 parasites/ field, 3 + : 1–10 parasites/ 10 fields, 2 + : 1–10 parasites/ 100 fields, 1 + : 1–10 parasites/ 1000 fields,0: 0 parasites per 1000 fields.

### Data analysis and interpretation

Epi- info version 3.5.1 was used to enter the data, which was subsequently uploaded to SPSS version 20 for analysis. Table and figure were used to provide descriptive statistics using frequency and percentage. The histogram, skewness, kurtosis, and Kolmogorov-smikirov test were used to verify the assumption of normality. In addition, the Levene test was employed to verify for variance homogeneity. For normally distributed data, mean and SD were used, while for skewed data, median and interquartile range (IQR) were used. For normally distributed and skewed data, respectively, the Paired t-test and Wilcoxon Sign Rank test were employed to compare mean differences in hematological profiles before and after treatment.

## Result

### Socio-demographic and clinical characteristics

From a total of 463 VL patients all most all (99.4%) study participants were males. The mean age of the study participants was 23 ± 7.32 years with a range of 13- 72 years. Majority 23.3% and 23.1% of the patients were graded as + 1 and + 2 respectively, with regard to parasite load. About 349 (75.4%) of the patients takes first line drug while the rest were treated with second line drug (Table1).

**Table 1:** Socio-demographic and clinical characteristics of visceral leishmaniasis patients at University of Gondar comprehensive specialized referral hospital leishmaniasis research and treatment center, Northwest Ethiopia2019

Variables	Frequency	Percent
Sex		
Male	460	99.4
Female	3	0.6
Age		
< 20 years	154	33.3
20–40	288	62.2
> 40	21	4.5
Site of aspiration		
Spleen	391	84.4
Bone marrow	72	15.6
Parasite load		
1 +	108	23.3
2 +	107	23.1
3 +	98	21.2
4 +	59	12.7
5 +	44	9.5
6 +	47	10.2
Date of completion of treatment		
Day 17	349	75.4
Day 28	114	24.6

### Hematological parameters of study participants

**Fig. 1:**
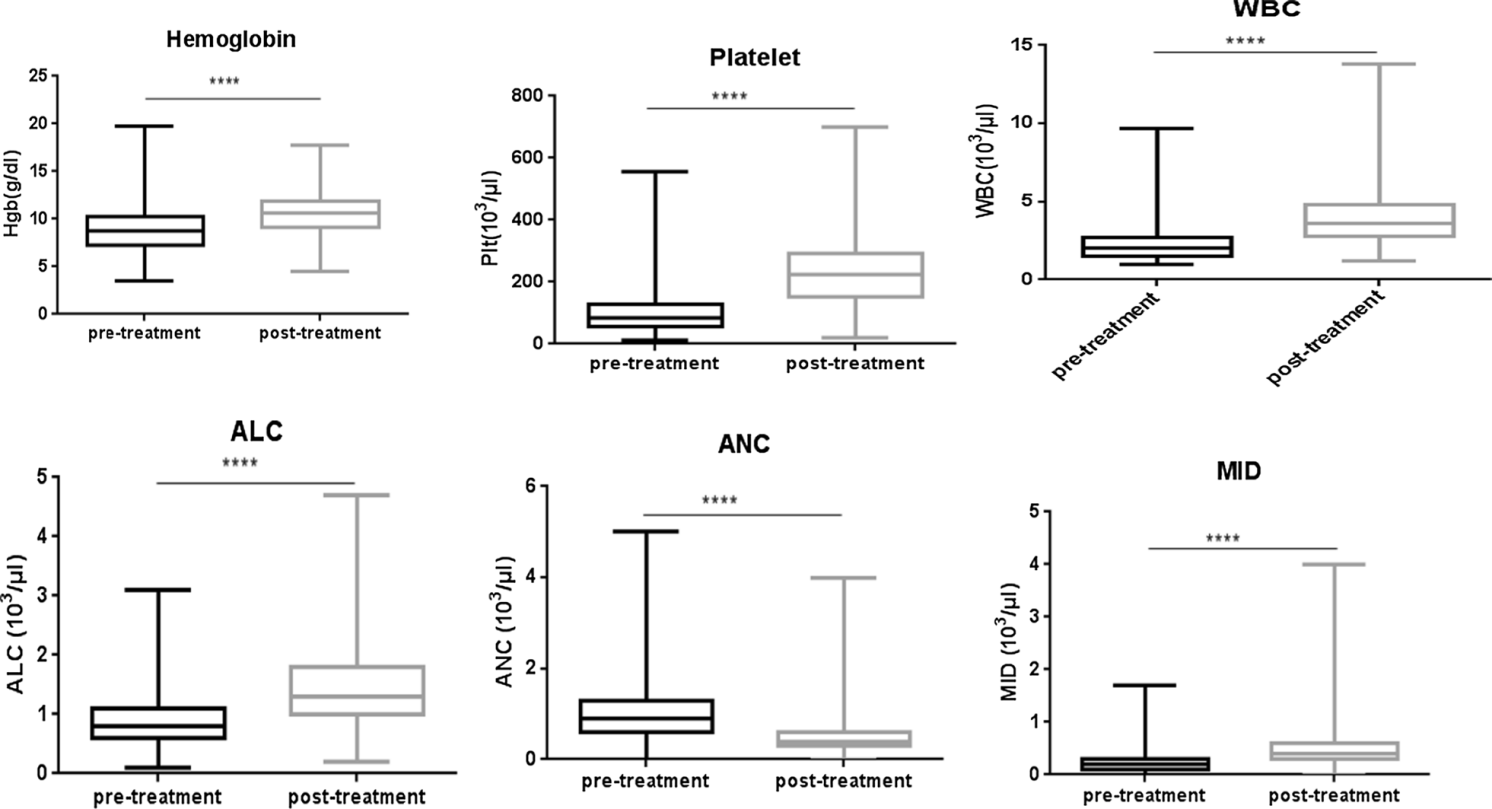
Pre and post treatment hematological parameters of VL patients (N = 463) at, University Gondar comprehensive specialized referral leishmaniasis research and treatment center Northwest Ethiopia, 2019. *WBC* white blood cell, *ANC* absolute neutrophil, *ALC* absolute lymphocyte, *MID* Absolute monocyte, basophil, and eosinophil

Parameters mainly Hb, Hct, MCHC RBCs, and platelet count were normally distributed whereas the rest were skewed. Post treatment mean RBCs count, Hb, Hct and Platelet count showed a significant increment compared to pre-treatment. On the other hand except ANC post treatment median values of WBCs, ALC, absolute mixed count, MCV and MCH showed a significant increment compared to pre-treatment (Table 2 and Fig. 1).

**Table 2:** The comparison of hematological parameters of study participants at University Gondar comprehensive specialized referral hospital leishmaniasis research and treatment center Northwest Ethiopia, 2019

Parameters	Pre-treatment	Post-treatment	P value
Total WBCs (10^3^/µl)	2.26 ± 1.04	3.94 ± 1.76	< 0.001
ALC (10^3^/µl)	0.94 ± 0.45	1.44 ± 0.65	< 0.001
ANC (10^3^/µl)	1.04 ± 0.71	0.48 ± 0.38	< 0.001
MID (10^3^/µl)	0.24 ± 0.19	0.48 ± 0.37	< 0.001
RBCs (10^6^/µl)*	3.35 ± 0.81	3.77 ± 0.83	< 0.001
Hb (g/dl)*	8.9 ± 2.22	10.5 ± 2.08	< 0.001
Hct (%)*	27.27 ± 6.49	32.2 ± 6.77	< 0.001
MCV (fl)	82.35 ± 8.63	85.49 ± 8.26	< 0.001
MCH (pg)	26.96 ± 4.11	28.05 ± 2.5	< 0.001
MCHC %*	32.5 ± 1.95	32.7 ± 2.77	0.116
Plt (10^3^/µl)*	97.75 ± 65.86	230.3 ± 109.4	< 0.001

*WBC* white blood cell, *ANC* absolute neutrophil, *ALC* absolute lymphocyte, *MID* Absolute monocyte, basophil, and eosinophil, *RBC* red blood cell, *Hb* hemoglobin, *Hct* hematocrit, *MCV* mean cell volume, *MCH* mean cell hemoglobin, *MCHC* mean cell hemoglobin concentration, *SD* standard deviation, *IQR* inter quartile range; *Indicates normally distributed data presented with mean and standard deviation. P-value, 0.05 considered statistically significant

Anemia was defined as a decrement in Hb concentration below the normal. The overall prevalence of pretreatment anemia was 85.5%. On the other hand the magnitude of leukopenia and thrombocytopenia before initiation of treatment was 83.4% and 75.8%, respectively. Likewise neutropenia and lymphopenia were commonly encountered among 84.4% and 60.3% of prior to treatment correspondingly (Table 3).

**Table 3:** Pretreatment hematological parameters of VL patients at, University of Gondar comprehensive specialized referral hospital leishmaniasis research and treatment center Northwest Ethiopia, 2019

Parameters	Results		Reference range*
	normal	Low	
WBCs	77 (16.6%)	386 (83.4%)	3.2–8.8
ANC	81(17.5%)	382(82.5%)	1.6–5.1
ALC	184 (39.7%)	279 (60.3%)	1.0–3.5
MXD	163 (35.2%)	300(64.8%)	0.2–1.0
RBCs	185 (40%)	278 (60%)	3.45–6.25
Hb	67 (14.5%)	396 (85.5%)	11.0–16.7
Hct	88(19%)	375(81%)	32.1–56.6
MCV	149 (32.2%)	314 (67.8%)	85–100
MCH	285 (61.6%)	178 (38.4%)	25.8–32.8
MCHC	460 (99.4%)	3 (0.6%)	28.5–34.4
Plt	112 (24.2%)	351 (75.8%)	128–432

*Indicate reference number

On the other hand the overall prevalence of post-treatment anemia, leucopenia and thrombocytopenia was 58.3%, 38.2% and 19.2% respectively. Neutropenia and lymphopenia was reported among 98.5% and 29.8% of the study participants, respectively (Table 4).

**Table 4:** Post-treatment hematological parameters of VL patients (N = 463) at, University Gondar comprehensive specialized referral hospital leishmaniasis research and treatment center Northwest Ethiopia, 2019

Parameters	Results		Reference range*
	normal	Low	
WBCs	286 (61.7%)	177 (38.2%)	3.2–8.8
ANC	7 (1.5%)	456 (98.5%)	1.6–5.1
ALC	325 (70.2%)	138 (29.8%)	1.0–3.5
MXD	364 (78.6%)	99 (21.4%)	0.2–1.0
RBCs	302 (65.2%)	161 (34.8%)	3.45–6.25
Hb	193 (41.7%)	270 (58.3%)	11.0–16.7
Hct	241 (52%)	222 (48%)	32.1–56.6
MCV	243 (52.5%)	220 (47.5%)	85–100
MCH	375 (81%)	88 (19%)	25.8–32.8
MCHC	461 (99.6%)	2 (0.4%)	28.5–34.4
Plt	374 (80.8%)	89 (19.2%)	128–432

*Indicate reference number

Moreover Box plots used to present pre and post treatment hematological parameters of VL patients.

### Correlation of parasite load with hematological profiles of VL patients

Correlation analysis had been carried out to assess the association of hematological profiles with the severity of disease. According to our result parasite load showed a significant negative correlation with both pre and post treatment hematological profiles mainly with pre-treatment WBCs count and ALC, in addition to post-treatment ALC, RBCs count and Hb concentration of VL patients (Table 5).

**Table 5:** Pearson and Spearman’s rank-order correlations of different hematological parameters with parasite load of the disease of VL patients at, University Gondar comprehensive specialized referral hospital leishmaniasis research and treatment center Northwest Ethiopia, 2019

Correlation coefficient	Parameters				
	Pre-WBCs	Pre-ALC	Post-ALC	Post-RBCs	Post-Hb
Rho	− 0.116*	− 0.204*	− 0.097*	− 0.103	− 0.12
P value	0.013	< 0.001	0.037	0.026	0.010

*Pre-WBC* pre-treatment white blood cell, *Pre-ALC* pre-treatment absolute lymphocyte, *Post-ALC* post-treatment absolute lymphocyte, *Post-RBC* post-treatment red blood cell, *Post-Hb* Post-hemoglobin

*Indicates spearman’s rank correlation and the rest are Pearson correlation for skewed and normally distributed data respectively

## Discussion

The goal of this study was to compare the hematological profiles of VL patients before and after anti-leshimania treatment. According to our findings; the bulk of the patients were males aged 20 to 40 years old. This could be because men are more likely to engage in outdoor activities, making them more vulnerable to sand fly bites. Furthermore, data indicate that in endemic locations such as Ethiopia, younger age groups are particularly vulnerable to the disease since they have not yet developed acquired immunity [4, 14].

Our result demonstrated that RBCs parameters mainly after treatment mean RBC count (3.77 ± 0.84 × 106/µl), Hb (10.5 ± 2.1 g/dl) and Hct values (32.2 ± 6.81) exhibited a substantial increment compared to pre-treatment one RBC count (3.34 ± 0.82 × 106/µl), Hb (8.9 ± 2.2 g/dl) and Hct (27.3 ± 6.5), respectively. The overall prevalence of pre-treatment anemia was 85.5%, which was similar to 83.8% [15] and Nepal 90% [16]. In contrast, our results were lower than those reported in prior studies from Gondar, where the prevalence ranged from 94.4% to 97.4% [17] and India 93% to 100% [18]. Furthermore, it was greater than studies from Yemen (59.6%) and New Delhi, India (64.8%) [19]. On the other hand the overall frequency of post-treatment anemia was 58.3%, which was lower than the prior report from Gondar, which had a prevalence of 93% [17]. The variance in prevalence between studies could be due to differences in overall sample size, threshold values used for anemia diagnoses, and the study's design.

Anemia can be caused by a variety of factors, including RBC sequestration and destruction in an enlarged spleen, immunological mechanisms, and changes in RBC membrane permeability in VL patients [10]. Besides amastigote, the parasite's intracellular stage proliferates in mononuclear phagocytic systems, where a large amount of iron is stored. A ligand on the surface of amastigotes binds hemin with high affinity and can also exploit iron from heme and Hb for nutritional purposes. This ligand may participate in intracellular heme transport, resulting in iron depletion for erythropoiesis [20]. Besides amastigote, the parasite's intracellular stage proliferates in mononuclear phagocytic systems, where a large amount of iron is stored. A ligand on the surface of amastigotes binds hemin with high affinity and can also exploit iron from heme and Hb for nutritional purposes. This ligand may participate in intracellular heme transport, resulting in iron depletion for erythropoiesis [20]. Furthermore, the parasite directly scavenges iron from macrophage iron pools, which is critical for them to avoid oxidative stress in the host, as iron is a cofactor for the antioxidant enzyme superoxide dismutase (Fe-SOD). The inactivation of Fe-SOD has an impact on their virulence and intracellular survival [21].

The current study's findings revealed that the post-treatment mean platelet count (230 ± 109 × 10^3^/µl) increased when compared to the pre-treatment (97 ± 65 × 10^3^/µl). The pre-treatment thrombocytopenia was 75.8%, which was consistent with a previous report from Nepal (72.5%) [16]. However, it was lower than a Gondar report of 90.1% [9], Iran 88.8% [15] and India's range of 83.7% [22] to 85% [18] Furthermore, our result was higher than previous reports from Yemen 55.3% [8] and New Delhi, India 52.7% [19]. The discrepancy in the total prevalence of thrombocytopenia between studies could be explained by differences in total sample size, optimal cutoff value used for classifications of thrombocytopenia, and study design. In general, thrombocytopenia may be caused by bone marrow suppression and hepatomegaly as a result of disease progression. Furthermore, because one-third of platelets are stored in the spleen, abnormalities in this organ result in a decrease in platelet count [10].

In our study, the median post-treatment value of WBC parameters increased significantly when compared to pre-treatment. The overall prevalence of pre-treatment leukopenia was 83.4%, which was lower than studies from Gondar (95.4%) [9] and India (100%) [18]. On the other hand, it was higher than previous reports from India, which ranged from 37.4% [19] to 60.5% [22], Yemen 53.2% [8] and Nepal 67.5% [16]. The current study found that 60.3% of patients had pre-treatment lymphopenia, which was higher than the previous study in Gondar, which found 37.9% [9]. Furthermore, the current study found that 84.4% of patients had pre-treatment neutropenia, which was higher than previous reports from Yemen 49.9% [8] and Nepal 27.5% [16]. In contrast to the other hematological parameters, ANC was the only one that did not improve after treatment. The Turkey study yielded a consistent result. This could be related to pentavalent antimonials treatment, which could result in an increase in pancreatic enzymes and transaminases [23].

WBC parameters improved after treatment, with 38.2% and 29.8% prevalence of leukopenia and lymphopenia, respectively. Leukopenia may be associated with symptoms that last for a long time and are caused by splenomegaly. Furthermore, the leishmania parasite invades and multiplies in macrophages, potentially triggering an inflammatory response. During the acute and chronic phases of the inflammatory process, neutrophils and monocytes are the main players, with the involvement of other types of WBC that may be destructed as a result of the inflammatory process [10].

Furthermore, the current study's correlation analysis revealed that parasite load has a negative correlation with hematological parameters, specifically pre-treatment WBCs count, pre-treatment ALC, post-treatment ALC, post RBCs count, and post Hb concentration of the patients. Nepal had reported a consistent result [16]. As a result, a reduction in parasite load helps to improve hematological parameters. An improvement in hematological parameters after treatment with anti-leishmaniasis drugs may be associated with the impact of treatment through direct killing of the parasite inside the phagolysosome through inhibition of trypanothione reductase enzyme, an enzyme that recycles oxidized trypanothione to keep the trypanothione in a reducing state [24]. Furthermore, the development of Th1 will trigger a granuloma response, which is caused by the production of IFN-gamma and will aid in the resolution of the infection (7).

## Conclusions

The current study found that, with the exception of ANC, the rest hematological parameters of VL patients improved after treatment. Furthermore, correlation analysis revealed that parasite load had a negative correlation with these patients' hematological parameters. This could be an indication of the parasite's effect on these parameters, which could affect the patient's quality of life. As a result, we would like to encourage other researchers to conduct a further prospective study to rule out any potential factors that may be associated with it, particularly its association with neutrophil count.

## Limitations of the study

Retrospective nature of the study prevents us from establishing a cause and effect relationship.

## Strength of the study

The current study attempted to assess hematological parameters before and after treatment in areas where epidemiological data are limited and can provide comprehensive information, as the majority of previous studies attempted to depict pre-treatment hematological profiles of these patients.

## Data Availability

All data supporting the findings and conclusion are presented in the manuscript. Data that support the findings of this study are also available from the corresponding author upon reasonable request.

## Abbreviations

ALC: Absolute Lymphocyte Count
ANC: Absolute Neutrophile Count
Hct: Hematocrit
Hb: Hemoglobin
Fe-SOD: Superoxide Dismutase
MCH: Mean Cell Hemoglobin
MCHC: Mean Cell Hemoglobin Concentration
MCV: Mean Cell Volume
MXD: Mixed Cell Count (Eosinophil, Basophile and monocyte)
RBC: Red Blood Cell
VL: Visceral Leishmaniasis
WBC: White Blood Cell
WHO: World Health Organization

## References

[1] Leta S, Dao THT, Mesele F, Alemayehu G (2014). Visceral leishmaniasis in Ethiopia: an evolving disease. PLoS Negl Trop Dis.

[2] Hotez PJ, Kamath A (2009). Neglected tropical diseases in sub-Saharan Africa: review of their prevalence, distribution, and disease burden. PLoS Negl Trop Dis.

[3] Mehdi DS (2008). The effect of visceral leishmaniasis on some liver enzyme and blood parameter. J Thiqar Univ.

[4] Motuma K, Abera E, Wondu B, Negash A (2016). Visceral Leishmaniasis in Ethiopia: A Review. Eur J Biol Sci.

[5] El-Safi AES, Adm ASK, Hamza KM (2016). Hematological profile of patients with visceral leishmaniasis at Al-Gaderf State—Sudan. Clin Med.

[6] Santos PLD, Oliveira FAD, Santos MLB, Cunha LCS, Lino MTB, Oliveira MFSD (2016). The severity of visceral leishmaniasis correlates with elevated levels of serum IL-6, IL-27 and sCD14. PLoS Negl Trop Dis.

[7] Middib MM, Mouktar FAA. Hematological changes including the immune system in patients with visceral leshmaniasis at Al-Muthanna governorate. J Babylon Uni Pure Appl Sci. 2014;4(22).

[8] Al-Ghazaly J, Al-Dubai W, Abdullah M, Al-Gharasi L (2017). Hematological characteristics of yemeni adults and children with visceral leishmaniasis could eosinopenia be a suspicion index?. Mediterr J Hematol Infect Dis..

[9] Tesfaye E, Fissehatsion K, Terefe B, Enawgaw B (2017). Haematological Abnormalities in Visceral Leishmaniasis Patients Attending Gondar University Hospital; Retrospective Study. IJHPEBS.

[10] Varma N, Naseem S (2010). Hematologic changes in visceral leishmaniasis/kala azar. Indian J Hematol Blood Transfus.

[11] Ethiopia Ministery of Ethiopia (2013). Guideline for diagnosis, treatment and prevention of leishmaniasis in Ethiopia.

[12] Central Statistical Agency (2008). The 2007 population and housing census of Ethiopia.

[13] Yalew A, Terefe B, Alem M, Enawgaw B (2016). Hematological reference intervals determination in adults at Gondar university hospital, Northwest Ethiopia. BMC Res Notes.

[14] Leta S, Dao THT, Mesele F, Alemayehu G (2014). Visceral leishmaniasis in ethiopia: an evolving disease. PLoS Negl Trop Dis.

[15] Sarkari B, Naraki T, Ghatee MA, Khabisi SA, Davami MH (2016). Visceral leishmaniasis in Southwestern Iran: a retrospective clinico-hematological analysis of 380 consecutive hospitalized cases (1999–2014). PLoS ONE.

[16] Agrawal Y, Sinha A, Upadhyaya P, Kafle S, Rijal S, Khanal B (2013). Hematological profile in visceral leishmaniasis. Int J Infect Microbiol.

[17] Mulaw T, Tariku A, Tsegaye AT, Abebe Z (2018). Effect of iron-folic acid supplementation on change of hemoglobin among visceral Leishmaniasis patients in northwest Ethiopia: a retrospective follow up study. BMC Hematol.

[18] Chufal SS, Pant P, Chachra U, Singh P, Thapliyal N, Rawat V (2016). Role of haematological changes in predicting occurrence of leishmaniasis-a study in kumaon region of Uttarakhand. JCDR..

[19] Gupta N, Kamla K, Mirdha BR (2017). Clinical and laboratory analysis of patients with leishmaniasis: a retrospective study from a tertiary care center in New Delhi. Iran J Parasitol.

[20] Carvalho S, Cruz T, Santarém N, Castro H, Costa V, Tomás AM (2009). Heme as a source of iron to Leishmania infantum amastigotes. Acta Trop.

[21] Das NK, Biswas S, Solanki S, Mukhopadhyay CK (2009). Leishmania donovani depletes labile iron pool to exploit iron uptake capacity of macrophage for its intracellular growth. Cell Microbiol.

[22] Saurabh K, Ranjan S, Prasad RR (2017). Clinical and haematological parameters associated with patients of visceral leishmaniasis in a district of North Bihar. Int J Community Med Public Health.

[23] An I, Harman M, Esen M, Çelik H (2019). The effect of pentavalent antimonial compounds used in the treatment of cutaneous leishmaniasis on hemogram and biochemical parameters. Cutan Ocul Toxicol.

[24] Singh OP, Singh B, Chakravarty J, Sundar S (2016). Current challenges in treatment options for visceral leishmaniasis in India: a public health perspective. Infect Dis Poverty.

